# COVID-19 testing experiences and attitudes among young adults and socially isolated older adults living in public housing, New York City (2022)

**DOI:** 10.3389/fpubh.2025.1484473

**Published:** 2025-06-23

**Authors:** Emily Gill, Zora Hall, Lorna E. Thorpe, Natasha J. Williams, Elle Anastasiou, Stefanie Bendik, Malcolm Punter, Jeremy Reiss, Donna Shelley, Marie Bragg

**Affiliations:** ^1^Department of Population Health, NYU Grossman School of Medicine, New York, NY, United States; ^2^Institute for Excellence in Health Equity, NYU Grossman School of Medicine, New York, NY, United States; ^3^Harlem Congregations for Community Improvement, New York, NY, United States; ^4^Henry Street Settlement, New York, NY, United States; ^5^Department of Public Health Policy and Management, NYU School of Global Public Health, New York, NY, United States

**Keywords:** COVID-19, public housing, testing intentions, information sources, health belief model

## Abstract

**Background:**

As part of an initiative to increase COVID-19 testing uptake among underserved populations, we conducted focus groups to explore experiences and attitudes related to testing in two understudied groups—young adults and socially-isolated older adults—recruited from residents living in New York City Housing Authority (NYCHA) public housing developments.

**Materials and methods:**

In June through November 2022, we conducted eight virtual focus groups with 21 young adults and 11 older adults living in NYCHA (*n* = 32 total). To identify themes, we conducted a rapid qualitative analysis approach.

**Results:**

Residents discussed four overarching themes: (1) trusted COVID-19 information sources; (2) reasons for testing; (3) barriers to testing, and (4) strategies to increase testing uptake. Findings were similar across the two age groups; both cited multiple sources of information, including major media outlets, government or public health officials, and doctors. Young adults were more likely to access information from social media despite concerns about misinformation. Participants identified several barriers to testing, such as long lines, insurance coverage, and cost. Young adults reported that at-home COVID testing was more convenient, while older adults expressed concern about accuracy and administering the tests themselves. Recommendations for improving testing emphasized easier access via a central well-known location, in-home visits, free or low-cost tests, and increased outreach.

**Conclusion:**

Mainstream media, doctors and public agencies remain the most trusted sources of information among younger and older residents alike. Many resident recommendations involved leveraging NYCHA infrastructure, highlighting the continuing role public housing authorities can play in COVID-19 and other health initiatives.

## 1 Introduction

To mitigate the effects of COVID-19, diagnostic testing is an essential tool to rapidly identify cases, isolate and treat infected individuals when indicated, and quarantine those who may have been exposed (1, 2). For diagnostic testing to be effective, tests must be widely available, easy to access, and trusted (3). Moreover, testing and access to other public health resources should be prioritized for communities facing increased vulnerability to the effects of the pandemic.

Within the United States, Black and Latinx populations have been disproportionately impacted by COVID-19 infections, illness, and death—in part, due to fewer resources to help prevent the spread of the virus (4–7). Additional upstream drivers of these disparities include lower household incomes, higher propensity for working in essential services, as well as residing in high-poverty neighborhoods and overcrowded housing (8–11).

As part of the National Institutes of Health’s Rapid Acceleration of Diagnostics – Underserved Populations (RADx-UP) initiative, NYU’s Grossman School of Medicine and Global School of Public Health, in partnership with community-based organizations (CBOs), received funding to study strategies to improve COVID-19 testing among residents of public housing. New York City Public Housing Authority (NYCHA) is the largest public housing authority in the nation. Home to approximately 400,000 people, or roughly 5% of the city’s population, residents of NYCHA have an average household income of just under $25,000 and approximately 90% identify as Black or Latinx (12–14). With respect to transmission risk, residents are disproportionately employed in essential service sectors that were associated with higher COVID incidence and mortality rates, including healthcare, retail and food services, transportation, warehousing, and manufacturing (15–17). In addition, NYCHA developments have a population density that far surpasses that of the city as a whole (18–20).

Nested within NYCHA communities are two subgroups with distinct risk profiles: young adults and older adults. Young adults, though at a lower risk for COVID-19 morbidity and mortality, consistently experience the highest rates of transmission, and are disproportionately responsible for COVID resurgences and hotspots, making them a key group to target for preventative efforts (7, 21). Older adults, in contrast, are far more susceptible to the effects of COVID-19. The risks are compounded for older residents of NYCHA, who have poorer self-reported health as well as higher rates of chronic conditions and limitations on activities of daily living when compared to older residents living in New York City (NYC) (22). Moreover, the proportion of older adults who live alone is far higher in NYCHA, making this a particularly hard-to-reach group for health promotion and disease prevention services (22).

This paper presents findings from focus groups conducted in 2022, after the introduction of at-home testing. We focused on young adults (ages 16 to 29 years) and older adults (ages 60 years and older) because previous research has not focused on comparing these two groups’ knowledge and attitudes about COVID-19 testing options, potential barriers and facilitators to getting tested, reasons for testing oneself, and sources of information about testing. Though both groups are disproportionately impacted by COVID-19, they are distinct populations with distinct experiences, beliefs and needs. By highlighting where the two populations overlap versus where they may require separate, targeted interventions, these findings can be used to guide policies and programs that optimize reach across risk groups.

## 2 Materials and methods

### 2.1 Study setting and recruitment

From June through November 2022, we conducted eight virtual focus groups (via HIPAA Zoom) with 32 individuals. All focus groups were conducted in English. We focused recruitment efforts on NYCHA developments across three neighborhoods (Central Harlem, Lower East Side and East New York) in NYC, each chosen for their high public housing density and persistently high levels of COVID-19 infections. Individuals were eligible for study participation if they were residents of NYCHA who were either young adults (those 16–29 years old), or socially isolated older adults (those 60 years and older who met one or more of our pre-determined social isolation criteria, as seen in Table 1). Participants were referred to the study through community partners, who recruited individuals using flyers, social media, and one-on-one conversations with people who might have more limited access to virtual forms of communication. Community partners also conducted home visits to reach residents with limited mobility. Additional information on this academic-community partnership can be found in a previous publication (23). Once an individual was referred to the study team, they were screened, consented, and given the necessary information to join the focus group. Each focus group lasted approximately 1 h, and participants were compensated $30 for their time. The study protocol was approved by the Institutional Review Board of NYU Grossman School of Medicine (i20-01636).

**Table 1:** Participant characteristics.

Variable	Older adults (*N* = 11)		Young adults (*N* = 21)	
	*N*	Mean (SD) or %	*N*	Mean (SD) or %
Age		64.7 (5.8)		22.8 (4.6)
Ethnicity				
Hispanic or Latino	0	0.0	4	19.0
Not Hispanic or Latino	11	100.0	14	66.7
Unknown/Not reported	0	0.0	3	14.3
Race				
American Indian/Alaska Native	0	0.0	0	0.0
Asian	0	0.0	2	9.5
Native Hawaiian or Other Pacific Islander	0	0.0	0	0.0
Black or African American	11	100.0	13	61.9
White	0	0.0	0	0.0
More than one race	0	0.0	4	19.0
Unknown/Not reported	0	0.0	2	9.5
Gender				
Female	10	90.9	12	57.1
Male	1	9.1	6	28.6
Non-binary	0	0.0	2	9.5
Other	0	0.0	1	4.8
Social isolation				
I live alone without any other adults or children in the home	3	27.3	Not asked in young adult survey	
I am homebound	2	18.2		
I have limited mobility	7	63.6		
I have limited social interactions	7	63.6		
I have a physical disability that prevents me from leaving the house	4	36.4		

### 2.2 Semi-structured interview guides

The interview guide was informed by the Integrated Behavioral Model (IBM), which integrates two earlier social science theories—the Theory of Planned Behavior and the Theory of Reasoned Action (24). Following this framework, interview questions assessed residents’ general experience of the pandemic, their knowledge of and overall attitudes toward COVID-19 testing, who they trusted for COVID related information (normative referents), as well as barriers to testing and suggestions as to how the process can be improved (personal agency) (24). We also explored residents’ attitudes toward municipal agencies (e.g., NYCHA; NYC Department of Health and Hygiene) with the rationale being twofold. First, municipal agencies played a significant role in developing and maintaining the infrastructure for COVID-19 testing. Second, based on our earlier work, it may be difficult for agencies to establish and maintain trust among community members they serve (25).

### 2.3 Data analysis

Study team members reviewed responses after each focus group to preliminarily discuss findings and assess if new data were emerging. In total, five focus groups were conducted with young adults, and three with older adults, at which point no novel responses were generated and thematic saturation was declared. Once data collection was complete, we analyzed focus group data using rapid qualitative analysis methods (26, 27). Five study team members (1) developing a mix of deductive and inductive codes, (2) independently coding all transcripts after they were de-identified, and (3) comparing emerging themes, including some not detailed previously in the interview guide. After successive iterations, two members merged codes and generated themes about residents’ experiences with testing and other preventative behaviors. Where conflicts arose, coders reviewed transcripts together until consensus was achieved. We then used this template to create a matrix of themes, subthemes, and illustrative quotes.

## 3 Results

### 3.1 Participant characteristics

Participant characteristics are summarized in Table 1. Thirty-two adults participated across 8 focus groups. The mean age for a participant in the young adult age group was 22.8 years (SD = 4.6). Within this group of 21 adults, 62% identified as Black or African American, 19% identified as Hispanic or Latino, 10% identified as Asian, and 19% identified as more than one race. More than half (57%) of participants were female. The older adult age group was comprised of 11 participants, all of whom identified as Black or African American. The mean age of this group was 64.7 years (SD = 5.8), and almost all (91%) participants were female.

### 3.2 Overarching themes

Residents discussed four primary themes: (1) trusted COVID-19 information sources; (2) reasons for testing; (3) barriers to testing, and (4) strategies to increase testing uptake. To facilitate comparisons between the two study populations we organized results to highlight areas in which older adults and younger adults reported similar responses and ways in which they differed. Illustrative quotes can be found in Table 2.

**Table 2:** Major themes from focus group analysis.

Group	Theme	Sub-theme	Quote
Both age groups	Information sources	Major media outlets	“I listen to the news and I look at the rate of where’s the COVID at, [which] states and what part, and how it escalates.” (OA FG1) “I watch New York One. Sometimes I watch The View. Stuff like that.” (OA FG3) “I go to most of all those news from Twitter ‘cause there are daily updates from the World Health Organization on how the disease is being spread, the way it’s spreading, the rate at which it’s spreading also. From there, the likes of BBC, Fox News, CNN.” (YA FG2) “Sources I trust the most…would usually be from main articles from a reputable source. Like the New York Times, or Daily Mail, or any like—basically, I forget the name. Any social media pages that’s actually like a reputable source, pretty much.” (YA FG4)
		Doctors	“I do not know who to trust. If I really wanna know something, I’ll ask my doctor, and he’ll call me back. He’ll know, and tell me what I wanna know.” (OA FG1) “I definitely got my information…from my school. Also my doctor, he told me to go [get tested and vaccinated] and so I did.” (YA FG1)
		Government	“Earlier in the pandemic, I was so grateful for [the governor] and the mayor and all of them, ‘cause it was just so informative. ‘Cause they was all together, spreading information properly. It did not make you confused.” (OA FG1) “Another source I would say would be like the CDC website… Some of the posts were on Facebook, people would screenshot and repost stuff that was on the CDC website so that everybody else could be aware of what was going on at the time.” (YA FG4)
	Reason for testing	Known COVID exposure	“Well, I know I would go get tested if I know I’ve been around somebody who got [COVID].” (OA FG1) “The reason I actually got tested recently was because I came into contact with someone who tested positive for COVID, and I did not feel safe. I just had to go test.” (YA FG2)
		Symptomatic	“If I catch a cold and I have a cough, I make sure I go get tested.” (OA FG2) “Whenever I see any kind of symptoms in my family, whether it’s fever, cough, shortness of breath, anything, we would get tested.” (YA FG2)
	Barriers to testing	Cost	“If you do charge [for tests], you know a lot of people ain’t gonna pay. They ain’t gonna have it to pay… a lot of people, you do not even have to be homeless, a lot of people just cannot pay.” (OA FG1) “Is testing free? ‘Cause I know for some people who do not have insurance, that’s probably a barrier. Getting testing would probably give them some sort of charge.” (YA FG1)
		Long lines	“At a site, sometimes you go and the line be so long. I cannot be doing all that standing on my knees. Uh-huh, I cannot be doing that, my knees, my back.” (OA FG1) “It was really hard trying to get a COVID test because of actual there not being enough time for the testing site to take people, and there being too many people wanting to get tests.” (YA FG1)
		Bureaucracy	“I do not go to every testing site. Some of them ask for your medical card; some of them ask for your insurance card. Some of them just ask for your ID. I do not understand.” (OA FG1) “I tried to go get tested because I felt sick, but come to find out I could not get tested because my dad switched my Medicaid over to his. He lives in a different state so that stopped my mom from doing what she had to do, and they could not see me.” (YA FG1) “I know there’s some places that would not take my insurance, and then it would be complicated. Like, ‘Oh, they are gonna send a bill,’ something like that. It might get paid for, it may not.” (YA FG4)
		Fear of result	“No, I’m not gonna keep [testing], ‘cause that’s letting COVID get in my head.” (OA FG1) “A lot of people do not like to be tested. They do not wanna be tested. A lot of people do not wanna know… I think they afraid to find out that they have it.” (OA FG1) “There’s also that reasoning of not wanting to get a positive result and having to isolate. I think, for some people, that might be scary, and it’s easier to just—I guess, if you do not see it, then it’s not real, like if you do not get a positive result, then it’s not real, you do not have COVID.” (YA FG2)
	Strategies and suggestions to improve testing	Central location	“I think maybe if [testing] was in NYCHA and in the community centers…I think that would help because people would know they did not have to go far. It’s in their neighborhood. They can go right there.” (OA FG2) “I feel like (19) should have maybe a actual room or place where they are like, ‘Hey, you could get tested here Tuesdays, Wednesdays, Thursdays, or Mondays, Wednesdays, Fridays, or on the weekends if you cannot.’ …Again, with convenience because now, it’s in the NYCHA building, so it’s like, ‘I just got a go downstairs and get tested. Now, I do not have an excuse for not going.’” (YA FG5)
		In-home visits	“I would tell NYCHA, if they wanna try to help us, especially the ones like myself included—I have knee problems… I think they should do a home visit. I feel that I’m exposed by going out and trying to go to one of those pop-up clinics… I feel I’m exposing myself. The other thing I think if they came and made a home visit, okay, I know you coming. I can mask up, you mask up.” (OA FG1) “I actually do think that it would be nice if we can get tested at home, like a professional can come over to our house on demand and then get us tested.” (YA FG2)
		Distribution of test kits by NYCHA	“Yeah, they could put [test kits] in a bag and put it on the door. Just put it on the doorknob.” (OA FG1) “I feel like at least five COVID test per resident who lives in the apartment would be beneficial… I feel like, even if they do not accept it, they just have it. Even if they do not want it, they just have it. I feel like, maybe, putting it inside the mailbox would be good… Also, maybe setting up a date and an event where they are gonna be handing out masks and COVID tests and any other supplies that they think could be beneficial.” (YA FG3)
		Free or very cheap tests	“I do believe that we should receive the free kits at least once every three months, and we can test ourselves so we can be aware and continue doing whatever other protocol we need to do.” (OA FG1) “The U. S. government should try to make home [test] kits available to people…I do not mean the one that has the very high price on it. They should bring it down to people’s level where everyone from the low class to the high class could easily afford it and get it.” (YA FG2)
		Increased outreach	“What they need to do is probably put it in the center, call us up or put a thing on the wall, letting us know we can go and take a test or something. Or have (19) call or text us or something, telling us.” (OA FG1) “Nobody that I know of [is testing]. I do not even think that they are aware that they can go to the community center and go get it. When I first saw the table with the COVID for NYCHA… they are the ones that told me that where to go get it. They said, ‘It’s in your area. Make it available to you.’ I would’ve never known that if they wasn’t there.” (OA FG3) “Probably just more thoroughly reach out to people. I did not get my home testing. Honestly, I did not even know that was a thing that was happening. If I knew, I probably would a got some.” (YA FG1)
Older adult group	Local sources of information	Faith-based organizations	“My church—we have information every other Thursday, and we also do testing. We also give out masks and let people know how often they should be tested, and they should be consistent about getting tested.” (OA FG2)
	Reason for testing	General social exposure	“Since I know I do go out, I do pass people. I try to stay out of a crowd, but I do wanna know.” (OA FG1) “Every now and then when I know I’m out and about. That’s usually the time when I get tested.” (OA FG3)
		Routine	“Let us say, I have not got tested in a couple of weeks or so. I see the tent, I’ll be like, “You know what, I have not got tested in a couple of weeks.” (OA FG1) “At least once a month, I go to get tested. Whenever they are there, I get tested. I make sure I get tested often.” (OA FG2)
	Barriers specific to at-home testing	Lack of accessible instructions	“One person brought about five kits to me. She said, “I do not know what to do with this. They just gave it to me, and I do not know how to use it.” That is really what’s happening when they giving you these kits. They do not show you what to do. Most of the seniors have a problem reading and seeing. Even with me myself, I have glaucoma. I cannot see out a one of my eyes.” (OA FG2)
		Accuracy	“That’s my concern that is it—am I doing it right, or is it as accurate as getting it done by professionals?” (OA FG2)
Young adult group	Social media as a primary conveyer of information	Timeliness	“I get my information from my doctor and friends as well, but majorly from social media because, as a platform I feel like information is spread quickly” (YA FG2)
		Concerns about accuracy	“I get information from social media, but sometimes I really do not trust it because anybody could put anything on the internet. I’d rather go to the source.” (YA FG4) “I see a lot of updates on Twitter, but I do not know how accurate that is, because on social media they like to tweak information just to get a lot of people to look at their stuff.” (YA FG5)
	Reason for testing	Protecting others	“I played a lot a sports, so just making sure I was safe and keeping my teammates safe.” (YA FG1) “I’m not really afraid of getting tested. I’d rather know that I have it and, God forbid, like I’ll be able to prevent the spread, and I’ll be able to isolate. I do not wanna get people sick.” (YA FG4)
	Barriers specific to on-site testing	Location	“I think testing is huge, making it more accessible, closer to us because my testing center is a 20 min walk, at least.” (YA FG2) “Some people live far from certain pharmacies or certain places that do COVID testing. They just cannot make it.” YA FG1
		Inconvenience	“I feel like people will get tested more if they could be getting tested at home because convenience is everything. You do not know if these people have kids. You do not know how busy their schedule is for them to actually sit at a City MD, at a pharmacy, or whatever to wait to get tested a couple hours, wait in some line to then figure out that they do not got COVID at all. Like, ugh, the frustration, the waste of time is just outrageous.” (YA FG5)
		Discomfort	“I really liked the option…of having the home test now, so I’m not so paranoid about them digging all in there. I could do it myself at my own pace.” (YA FG4)

OA, Older Adult; YA, Young Adult.

#### 3.2.1 Sources of information

Participants from both age groups cited major media outlets, doctors, and government agencies as trusted information sources. Regarding the differences in themes between the two groups, younger adults mainly accessed this information using social media, including Twitter, Facebook, and TikTok, though they noted a risk of misinformation on these sites and made efforts to identify the original source to verify the information. In contrast, older adults reported receiving information mainly from television news, and described local government sources (e.g., governor and mayor) as important. Only the older adults cited faith-based organizations as a source of information and materials related to COVID-19. Both groups included doctors as an important source of information and guidance. Participants mentioned major media outlets as useful for monitoring the spread and general risk level of COVID-19, though none mentioned using these outlets as a resource for receiving the most up-to-date recommended public health practices. Of note, some participants referenced government as also being a source of confusion, stating that communication was better in the earlier stages of the pandemic, because information was *“coming from one office at one time” (OA FG1).* Moreover, some participants cited the government as propagating the norm that COVID-19 precautions were no longer necessary (YA FG5).

#### 3.2.2 Reasons for testing

Having a recent confirmed COVID-19 exposure and experiencing COVID-19 symptoms were the two main reasons both groups cited as to why they would decide to get tested or have been tested previously. However, only older adults indicated that they intended to test because of general exposure to others, regardless of (known or unknown) COVID-19 status of the people they interacted with. Young adults frequently cited the safety of others as motivation for testing themselves (YA FG2). Additionally, some participants in the older adult group indicated they were testing themselves routinely, often within a defined window of time they deemed appropriate, which could range from 2 weeks to 4 months (OA FG2-3).

#### 3.2.3 Barriers to testing

Participants across focus groups cited cost, bureaucracy (e.g., being unsure of insurance requirements or coverage), long lines, and fear of a positive test result as barriers to COVID-19 testing. Most participants indicated that requiring any payment for tests would significantly decrease the likelihood that they would get tested (YA FG4). Participants also described multiple reasons why long lines and wait times were barriers, including mobility issues (e.g., pain or difficulty with standing), having to take time off from work, fear of exposure while waiting in line, logistical difficulties for those with children, and general negative feelings that arise from long wait times, including anxiety. Both groups indicated that fear of a positive test was also a barrier. Many people preferred not to know their COVID-19 status (in some cases even when individuals were symptomatic) because receiving a positive result meant lost wages, the need to notify others, possible medical care, and the potential mental and emotional toll of having to isolate from others. As for the older adult group, some expressed the opinion that at-home testing presented more barriers than on-site testing. Among these concerns were a lack of confidence in their ability to self-administer a COVID-19 test and a lack of confidence in the accuracy of the result (OA FG1). Conversely, considering the barriers they cited regarding on-site testing, the young adult groups expressed a preference for at-home testing. A specific concern with on-site testing in this age group was inconvenience, citing the time spent traveling and waiting in line as barriers, as well as the pain and discomfort that can come from having another person swab your nasal cavity. To them, at-home testing allowed for autonomy over both issues.

#### 3.2.4 Suggestions and strategies to increase testing uptake

Despite age differences and some divergence in testing motivation and preferences, there was considerable consensus on strategies and suggestions to improve testing. Both groups suggested opening test sites in central locations to minimize inconvenience. Location suggestions included NYCHA-affiliated management offices and community centers, as well as high-traffic non-NYCHA areas outside of major business or grocery stores. Participants expressed support for maintaining or restoring the placement of pop-up and mobile testing sites. Older adults proposed in-home testing administered by a professional, as a solution to address both mobility issues experienced by older adults and their concerns about self-administering the tests. Participants in the young adult focus groups were also supportive of this idea, though they expressed some concerns about feasibility, given the volume of individuals needing tests and risk of infection for any health officials entering the residents’ homes. Additionally, residents suggested that NYCHA offer test kits at no-cost, either by delivery or pick-up within the building. Universally, participants believed that providing free or affordable test kits was the role of governmental or municipal agencies. Finally, participants proposed greater outreach as a strategy to increase testing. Often participants noted that they or others would be willing to test but aren’t aware of their options. Flyers placed in common areas and under apartment doors, text messages, phone calls, social media and in-person events held at NYCHA community centers were some ideas for expanding reach.

## 4 Discussion

To our knowledge, this is the first study to identify barriers specific to at-home testing for older adults, and to draw comparisons between the attitudes of older adults and younger adults toward COVID-19 testing. We found that mainstream media, doctors and government agencies remain the most trusted sources of information among younger and older residents alike. Residents offered viable strategies to improve testing, including suggestions to leverage the infrastructure of public housing for testing services, and for public housing authorities to continue distributing tests to all residents.

Among the young adults and older adults who participated in age-specific focus groups, multiple themes emerged that spanned both groups. Both groups reported several trusted sources of information, including major media outlets, doctors, and public health officials or government employees. While this is consistent with previous literature (28–32), it is notable these remained the named trusted sources well into the pandemic, despite widespread concerns about erosion of trust in public health authorities and the medical establishment, and of the proliferation of fake news (28, 30, 33–35). One of the main differences between the groups was that young adults were more likely to access information from social media, despite being aware that these sources may contain misinformation, a concern noted in prior COVID-19 studies (25, 30, 32). Conversely, older adults preferred people with whom they can actually discuss their health, as opposed to the Internet (36). This included healthcare providers, friends and family, and community or faith-based organizations.

Both groups cited a COVID-19 exposure or exhibiting symptoms as the main reason for getting tested. Separately, older adults emphasized awareness of their own vulnerability to the virus in high-risk settings (e.g., social events) as a reason for testing. This is consistent with other studies examining the relationship between older age and COVID-related behaviors (37, 38). Feeling more susceptible, older adults are more likely to test, get vaccinated, and more likely to perceive preventative behaviors—such as social distancing and avoiding crowds—as effective (37, 39, 40). In contrast, young adults in the study were motivated to test to protect older or more vulnerable people in their social circle. Younger adults, though they see themselves as less susceptible, recognized the increased risk to older groups, and often cited “protecting others” as a primary motivation for testing and vaccination (31, 41).

Participants identified a number of barriers to testing, including some of which have been identified in prior studies, such as long lines, difficulty navigating questions about insurance coverage and how to access tests, and cost (28, 30, 42–44). While at-home COVID testing was seen as a more convenient and accessible alternative to in-person testing by younger participants, older adults expressed concern about making an error during self-testing. As other population groups have identified, both young adult and older adult participants emphasized the benefits of easier access via a central well known location, in-home visits, free or low-cost tests, and increased outreach (28, 42, 45, 46). Notably, all groups recommended the housing authority as a venue for testing service provision.

Participants in this study provided recommendations that could be utilized and expanded on, many of which were specific to NYCHA. For example, participants proposed that building management could distribute PPE or test kits, and a number of participants reported a higher level of cleanliness in communal areas of their buildings (e.g., lobbies; stairwells), which supported their ability to reduce transmission of COVID-19 through safe hygiene practices. Though underexplored in research literature, there are examples of the critical role of public housing programs in addressing the spread and effects of COVID-19. One paper examining the impact of the U. S. Housing and Urban Development sponsored service coordinators found that throughout the pandemic these coordinators were crucial in several areas, including resident needs assessment, benefits and program applications, dissemination of public health recommendations, addressing loneliness/isolation, and coordination between organizations (47). In the face the of rapidly changing nature of the pandemic, as well as lapses in typical benefits and services, these coordinators were able to leverage their knowledge of residents’ needs, property resources and staff, and public and community service programs to great effect (47).

In NYC specifically, NYCHA was a key partner to government agencies and CBOs. Test & Trace – the city’s interagency initiative to provide testing, contact tracing, and aftercare support – implemented a mobile testing program to address testing inequities in neighborhoods disproportionately affected by COVID-19. Between June 2020 and December 2021, Test & Trace was the largest provider of COVID testing services in NYC, operating 586 testing sites throughout the city (48). Many of these testing sites were set up on or near NYCHA property, including on-site senior and community centers (48, 49). Moreover, 90% of NYCHA service coordinators surveyed indicated that their properties had offered on-site vaccination at least once (47). Our participants repeatedly referenced these on-site centers, stating that they were crucial in making testing and vaccination accessible, and should continue to serve as a site for COVID-19 related resources and services. One analysis of housing and COVID-19 found that living in NYCHA may have a protective effect (9). One possible explanation could be that as a public entity, NYCHA—and therefore the residents—can more easily be connected to public services and initiatives (49, 50). This was demonstrated in another study, whereby public housing residence was associated with lower rates of uninsurance and unmet medical need. The authors posited that housing stability and reduced housing costs may give residents the opportunity and resources needed to access care (51). Presumably, this holistic approach could also contribute to COVID-19 awareness.

Our study has some limitations, including the small sample size of 32 focus group participants. Focus groups were only conducted in English due to time and staffing constraints, which limited our reach to other populations, particularly Spanish-only speaking older adults who may have been linguistically isolated (51). Additionally, using a virtual platform for the focus groups may have been a barrier to participation for some older adults, possibly hindering their enrollment. The confluence of these two factors may explain why our older adult group was both smaller and more homogenous than that of young adults. However, our sample can still be considered representative with regards to other characteristics: all participants were residents of public housing, and there was a high degree of similarity for certain socioeconomic factors (income, percent employment, educational attainment) that remained consistent across race/ethnicity (12, 52). Moreover, though women were overrepresented in our participant pool, this is also reflective of NYCHA’s overall population, including older residents (12). The timing of the study may also present a limitation – focus groups were conducted from June through November of 2022, when the level of public concern for COVID-19 was declining (52). Some of our focus group questions asked participants to recall how they felt during the early stages of the pandemic, which may have introduced some recall bias (53).

Despite these limitations, our findings are still a major contribution to the literature due to our populations of interest. A focus on residents of public housing is important given the particular vulnerabilities of this group, as well as the potential for public housing infrastructure to provide resources to improve COVID-19-related information sharing and testing. Equally important is our focus on two high-risk, understudied age profiles. To adequately target these distinct populations, a comparative understanding of their needs, beliefs and behaviors will be required, and our findings can be used to inform interventions in the event of future COVID surges or similar epidemics. Though it has been several years since the acute phase of the COVID-19 pandemic, there remains a potential for COVID-19 resurgences due to new variants, waning immunity, low vaccination rates, and the relaxation of social distancing and other protective behaviors. As such, mitigation strategies specific to COVID-19 may still be needed in the future (54–56). Moreover, our results are also broadly applicable to other infectious diseases, in particular respiratory viruses such as influenza, RSV, streptococcus, and other coronaviruses, which, like COVID-19, are seasonally epidemic, disproportionately affect the same populations, and have multiple means of diagnostic testing (clinical and at-home) that can be leveraged to reduce spread and burden of disease (57–60).

## Data Availability

The original contributions presented in the study are included in the article/Supplementary material, further inquiries can be directed to the corresponding author.
