# Supplementary figures and images for: COVID-19 testing experiences and attitudes among young adults and socially isolated older adults living in public housing, New York City (2022)
Source: Front Public Health. 2025 Jun 23;13:1484473. doi: 10.3389/fpubh.2025.1484473 (PMC12230297; doi:10.3389/fpubh.2025.1484473)

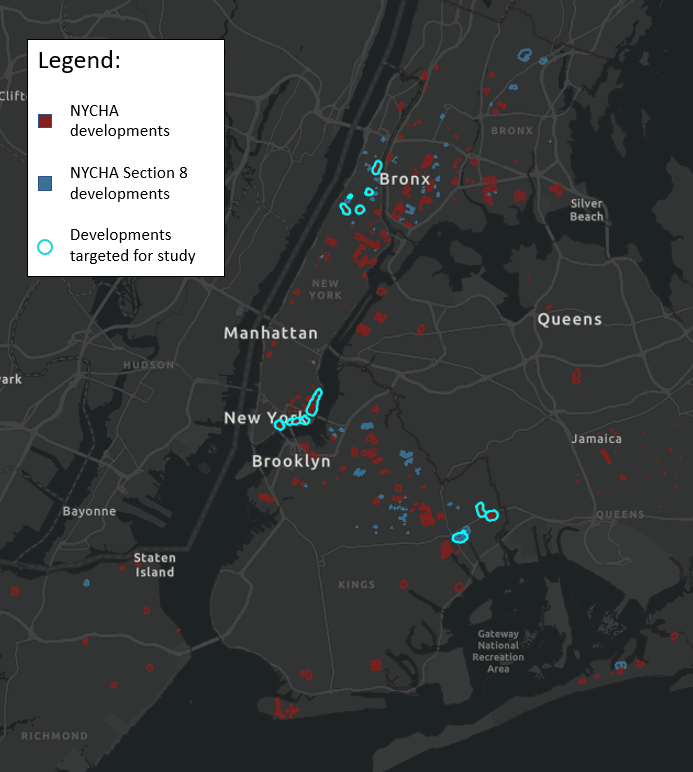

Supplement: Supplementary file 1 [file Image_1.png]
